# Supplementary material for: A Placebo-Controlled Trial of Cannabinoid Treatment for Disruptive Behavior in Children and Adolescents with Autism Spectrum Disorder: Effects on Sleep Parameters as Measured by the CSHQ
Source: Biomedicines. 2022 Jul 13;10(7):1685. doi: 10.3390/biomedicines10071685 (PMC9312464; doi:10.3390/biomedicines10071685)
Supplement: Supplementary file 1 [file biomedicines-10-01685-s001.zip › biomedicines-1784640-supplementary.pdf]

Supplemental Information

Title:

Impact of Cannabinoid Treatment on Sleep in Children and Adolescents with Autism Spectrum Disorder – A Double-Blind Placebo-Controlled Study

Aviad Schnapp[1,2], Moria Harel[1], Dalit Cayam-Rand[1], Hanoch Cassuto[3], Lola Polyansky[1], and Adi Aran[1,4*]

[1]Neuropediatric Unit, Shaare Zedek Medical Center, Jerusalem, Israel.

[2]Department of Pediatrics, Hadassah Medical Center, Jerusalem, Israel.

[3]Child Development Centers, Leumit Health Services, Israel.

[4]Hebrew University of Jerusalem, Faculty of Medicine, Jerusalem, Israel.

***Corresponding author:** Adi Aran, MD; Director, Neuropediatric unit, Shaare Zedek Medical Center

Tel: (+972) 508685034 • Fax: (972) 26416162. aaran@szmc.org.il

Supplementary methods: Interventions, Randomization and Blinding
Table S1. Inclusion and exclusion criteria for study participation
Table S2. Impact of cannabinoid treatment on sleep. Comparison of treatment effects in 2nd period
Table S3. Comparison of change in CSHQ scores (Mean ± SE) following the 1st and 2nd treatment that each participant received

Supplementary methods: Interventions, Randomization and Blinding

Interventions: All treatments were given as oil-based extracts for sublingual administration by drops containing 0.034 ml each (BOL pharma, Israel, FDA global G.A.P. approved).

Placebo solution was prepared to imitate the flavor and color of the whole-plant extract:

Material	Amount in % weight	Amount per unit (per ml)<br>Amount per drop (≈0.04ml)	Function
Olive oil	98.89%	0.9889ml /ml; 0.0395ml/drop	Solvent
Oleoresin oregano	1%	0.01ml / ml; 0.004ml/drop	Color and flavor masking
Raspberry o.s.	0.03%	0.003ml/ ml; 0.00012 ml/drop	Flavor enhancer
Paprika color	0.08%	0.008ml/ml; 0.00032ml/drop	Color

Pure cannabinoids were mixed with the placebo solution in the following ratios:

Material	Amount (W/W%)	Amount per unit (per ml)<br>Amount per drop (≈0.04ml)	Function
CBD	18.2% (182 mg/gram)	167 mg/ml; 6.7mg/drop	API
THC	0.91% (9.1 mg/gram)	8.3 mg/ml; 0.332mg /drop	API
Placebo solution	80.89% (Completion to 100% formulation)		Solvent

Whole-plant extract was mixed with the placebo solution in the following ratios:

Material	Amount (W/W%)	Amount per unit (per ml)<br>Amount per drop (≈0.04ml)	Function
CBD	18.2% (182 mg/gram)	167 mg/ml; 6.7mg/drop	WP
THC	0.91% (9.1 mg/gram)	8.3 mg/ml; 0.332mg /drop	WP
Placebo solution	80.89% (Completion to 100% formulation)		Solvent

The products were supplied to Shaare Zedek Medical Center in 6 batches (over 22 months of study duration). Certificate of Analysis from a certified, independent laboratory (Izun Pharma, Jerusalem, Israel) was supplied with each batch. Stability of the products was approved by the Israeli Ministry of Health to be 18 months at room temperature. Specification sheets of the products are available upon reasonable request. Surprisingly, given the low functioning of many participants and prior reports of reluctance to take other medications, only two participants were reluctant to take the intervention formulations.

Randomization and blinding

Eligible subjects were assigned an enrollment number in sequential order beginning with 01. Allocation to treatment arm was based on a randomization list. Randomization scheme was generated by BioStats Statistical Consulting Ltd. (an external biostatistical consulting firm). The blocked randomization was performed using SAS statistical software V9.4 via the plan procedure with a fixed block size.

The randomization scheme and lists were sent to the center for both periods of the study. The randomization list included sequential numbers and an assigned 3-letter code (several codes were assigned for each of the 3 treatment options). Randomization was kept at 1:1:1 ratio. The randomization list was encrypted by password and was sent prior to first dosing to the principal investigator.

The study whole-plant extract, the pure cannabinoids as well as placebo were packed in similar bottles and had similar color, taste and smell characteristics. The treatment products and placebo were both dissolved in olive oil. Addition of paprika color and oleoresin oregano to the placebo solution disguised the color completely. Addition of oleoresin oregano at high concentration was applied to mask the tastes of CBD and THC and raspberry flavor was added to mask the aroma (confirmed by a group of experienced tasters).

The code key was kept by BioStats Statistical Consulting Ltd. until study end. Neither the principal investigator nor any other team member or individual had access to the codes until study end. No unblinding occurred during the study.

Table S1. Inclusion and exclusion criteria for study participation

**Inclusion criteria**	1. Age 5 to 21 years old<br>2. A diagnosis of ASD according to the Diagnostic and Statistical Manual of Mental Disorders [Fifth Edition; DSM-5]<br>3. Moderate or greater behavioral problems as measured by a Clinical Global Impression Scale - Severity (CGI-S) score of 4 or higher at screening. Structured criteria were used to rate behavioral difficulties on the CGI-S, rather than overall ASD severity.
**Exclusion criteria**	1. Lifetime history of psychotic disorder<br>2. Current or former treatment with cannabinoids<br>3. A physical exam and laboratory results are out of the normal range for individuals with ASD, including a significant impairment in heart, liver, or renal function.<br>4. Any change in the pharmacological or behavioral treatment or change in the home or school settings (other than school holidays) in the 4 weeks prior to randomization or planned changes during the study.<br>5. Predicted low compliance to the study procedures (such as absence of a parent or caregiver able to consistently complete assessments throughout the study)

Table S2. Impact of cannabinoid treatment on sleep. Comparison of treatment effects in 2nd period

	**Placebo** n=35 [change in points]	**Pure cannabinoids** n=35 [change in points]	**Whole Plant** n=37 [change in points]	**Total** N= 107 [change in points]	***Sig***^
**Total CSHQ score**					
**Mean** ± SD	**0.4**±5.3	**-1.6**±5.0	**0.6**±4.9	**-0.2**±5.1	*0.14*
[median, range]	[0.8, -11.7 – 11.0]	[-1.3, -17.0 – 12.4]	[1.0, -12.0 – 12.2]	[0.0, -17.0 – 12.4]	
**Bedtime Resistance**					
**Mean** ± SD	**0.9**±2.0	**-0.3**±2.1	**0.1**±2.0	**-0.1**±2.0	*0.63*
[median, range]	[0.0, -6.0 – 5.0]	[0.0, -8.0 – 3.0]	[0.0, -4.0 – 4.0]	[0.0, -8.0 – 5.0]	
**Sleep Onset Delay**					
**Mean** ± SD	**0.1**±0.8	**0.0**±0.7	**-0.1**±0.9	**0.0**±0.8	*0.77*
[median, range]	[0.0, -1.0 – 2.0]	[0.0, -2.0 – 1.0]	[0.0, -2.0 – 2.0]	[0.0, -2.0 – 2.0]	
**Sleep Duration**					
**Mean** ± SD	**0.2**±1.5	**-0.4**±1.2	**0.3**±1.2	**0.0**±1.3	*0.05*
[median, range]	[0.0, -3.0 – 4.0]	[0.0, -4.0 – 2.0]	[0.0, -4.0 – 4.0]	[0.0, -4.0 – 4.0]	
**Sleep Anxiety**					
**Mean** ± SD	**-0.4**±1.4	**0.2**±1.1	**0.2**±1.3	**0.0**±1.3	*0.10*
[median, range]	[0.0, -4.0 – 3.0]	[0.0, -2.0 – 3.0]	[0.0, -4.0 – 3.0]	[0.0, -4.0 – 3.0]	
**Night Wakings**					
**Mean** ± SD	**-0.1**±1.2	**0.2**±1.2	**0.1**±0.9	**0.1**±1.1	*0.62*
[median, range]	[0.0, -3.0 – 4.0]	[0.0, -3.0 – 3.0]	[0.0, -2.0 – 3.0]	[0.0, -3.0 – 4.0]	
**Parasomnias**					
**Mean** ± SD	**0.2**±1.2	**0.0**±1.5	**0.1**±1.1	**0.1**±1.3	*0.81*
[median, range]	[0.0, -1.1 – 4.0]	[0.0, -3.5 – 3.9]	[0.0, -2.0 – 2.0]	[0.0, -3.5 – 4.0]	
**Sleep Disordered Breathing**					
**Mean** ± SD	**0.2**±1.0	**-0.3**±1.0	**-0.1**±0.9	**0.0**±1.0	*0.05*
[median, range]	[0.0, -1.0 – 5.0]	[0.0, -2.0 – 21.0]	[0.0, -3.0 – 2.0]	[0.0, -3.0 – 5.0]	
**Daytime Sleepiness**					
**Mean** ± SD	**-0.3**±2.3	**-0.5**±3.0	**-0.4**±2.3	**-0.4**±2.5	*0.93*
[median, range]	[0.0, -6.0 – 4.0]	[0.0, -9.0 – 5.0]	[0.0, -6.0 – 4.0]	[0.0, -9.0 – 5.0]	

Between-subject analyses of the change in the CSHQ scores following treatment in the 2nd 12-week treatment period. CSHQ: Children's Sleep Habits Questionnaire. Positive change (increment of CSHQ scores) indicates worsening of the sleep disorder. Change in the CSHQ scores from baseline following treatment is compared between the 3 treatment arms.

^One-way ANOVA for influence of treatments between study groups.

Notably, the difference between cannabinoid treatment and placebo was not statistically significant even when combining the two cannabinoid treatments into one group compared to placebo (data not shown).

Table S3. Comparison of change in CSHQ scores (Mean ± SE) following the 1st and 2nd treatment that each participant received.

	Group A (n=37)			Group B (n=35)			Group C (n=35)		
	1st treatment **Placebo**	2nd treatment **Whole-plant**	***Sig^***	1st treatment **Pure cannabinoids**	2nd treatment **Placebo**	***Sig^***	1st treatment **Whole-plant**	2nd treatment **Pure cannabinoids**	***Sig^***
**Total CSHQ score**	**-1.0**±1.0	**0.3**±0.8	*0.28*	**-3.0**±1.7	**0.4**±0.9	*0.10*	**-2.6**±0.9	**-1.4** ±0.8	*0.33*
Bedtime Resistance	**-0.3**±0.2	**0.0**±0.3	*0.45*	**-0.7**±0.5	**0.0**±0.3	*0.29*	**-0.4**±0.3	**-0.2**±0.3	*0.69*
Sleep Onset Delay	**-0.2**±0.1	**-0.1**±0.1	*0.43*	**-0.2**±0.1	**0.1**±0.1	*0.30*	**-0.1**±0.1	**0.1**±0.1	*0.39*
Sleep Duration	**-0.4**±0.2	**0.1**±0.2	*0.11*	**0.0**±0.3	**0.1**±0.2	*0.82*	**-0.6**±0.3	**-0.5**±0.2	*0.74*
Sleep Anxiety	**-0.4**±0.2	**0.1**±0.2	*0.12*	**-0.5**±0.2	**-0.3**±0.3	*0.63*	**-0.3**±0.3	**0.2**±0.2	*0.10*
Night Wakings	**-0.1**±0.2	**0.1**±0.2	*0.44*	**-0.6**±0.3	**0.0**±0.2	*0.07*	**-0.7**±0.2	**0.3**±0.2	*0.00*
Parasomnias	**0.0**±0.0	**0.3**±0.2	*0.89*	**-0.7**±0.4	**0.2**±0.2	*0.05*	**-0.5**±0.2	**0.1**±0.3	*0.09*
Sleep Disordered Breathing	**0.0**±0.2	**-0.1**±0.2	0.74	**-0.3**±0.2	**0.3**±0.2	*0.06*	**-0.2**±0.1	**-0.3**±0.2	*0.40*
Daytime Sleepiness	**0.5**±0.5	**-0.4**±0.4	*0.14*	**0.0**±0.7	**-0.2**±0.4	*0.76*	**-0.2**±0.5	**-0.5**±0.5	*0.67*

Change in CSHQ scores following treatment. Each participant received one treatment in a first 12-week treatment period and crossed-over to a different treatment in a second 12-week period. In each group, the change in scores in the first treatment period is compared to the change in scores in the second treatment period, comparing the two treatments that each participant received (a within subject analysis). CSHQ: Children's Sleep Habits Questionnaire. Positive change (increment of CSHQ scores) indicates worsening of the sleep disorder.

^Paired samples t-test for influence of treatments *within* study groups.
